# Beyond culturability: VBNC-like and differentially culturable *Mycobacterium tuberculosis* challenge growth-based interpretations of bacterial viability

**DOI:** 10.3389/fcimb.2026.1896879

**Published:** 2026-07-20

**Authors:** Jing Niu, Peng Zhang, Zihan Wei, Yuanwu Zou, Zhuo Wang

**Affiliations:** 1 Department of Clinical Laboratory, Xi’an Union Hospital, Xian, China; 2 Department of Clinical Laboratory, Shaanxi Provincial People’s Hospital, Xian, China; 3 Department of Clinical Laboratory, Shaanxi Provincial Hospital of Tuberculosis Prevention and Treatment Hospital, The Fifth People’s Hospital of Shaanxi Province, Xian, China

**Keywords:** bacterial viability, culturability, differentially culturable tubercle bacilli, *Mycobacterium tuberculosis*, VBNC-like phenotypes, viability continuum

## Abstract

Culturability remains the predominant method for assessing bacterial viability in tuberculosis research and clinical practice. However, growth-based detection provides only a limited and context-dependent dimension of *Mycobacterium tuberculosis* (MTB) survival. MTB populations may evade recovery by standard culture while retaining molecular, metabolic, or functional features inconsistent with irreversible cellular death, resulting in persistent discrepancies between culture outcomes and other biological or clinical indicators. This review explores viable but non-culturable (VBNC)-like phenotypes and differentially culturable tubercle bacilli (DCTB) as detection-defined frameworks for interpreting such growth-invisible populations. We analyze their methodological foundations and interpretative limitations, emphasizing that neither reflects a discrete or stable physiological state. Integrating evidence from resuscitation assays, molecular viability measurements, and clinical observations, we propose a continuum-based interpretation of MTB viability in which bacterial populations occupy diverse physiological states spanning survival potential and growth competence. Within this framework, apparent non-culturability is more accurately conceptualized as a conditional result of physiological limitations and methodological sensitivity, rather than as conclusive evidence of bacterial death or dormancy. This perspective provides a coherent conceptual foundation for interpreting bacterial persistence, clearance, and treatment response in tuberculosis.

## 1 Introduction

For more than a century, culturability has served as the dominant operational proxy for assessing bacterial viability in tuberculosis research and clinical practice (Rageade et al., 2014; Asmar and Drancourt, 2015; Heyckendorf et al., 2022). The ability of *Mycobacterium tuberculosis* (MTB) to generate visible growth on solid or liquid media has often been interpreted as evidence of viable bacilli and, in clinical contexts, as a marker relevant to treatment response and potential ongoing disease activity. Conversely, failure to recover MTB by standard culture has frequently been used as a surrogate for bacterial clearance (Horne et al., 2010; Phillips et al., 2013). This culture-dependent framework has influenced diagnostic standards, guided treatment monitoring, and informed regulatory endpoints. However, accumulating evidence from *in vitro* stress models, animal infection systems, and patient-derived specimens indicates that culture negativity does not necessarily reflect irreversible bacterial death. MTB populations may persist in culture-negative states while retaining molecular, metabolic, or functional features inconsistent with loss of viability (Mukamolova et al., 2010; Salina et al., 2014; Hu et al., 2016). These observations challenge the assumption that culturability is an intrinsic determinant of viability and instead suggest that it is a conditional outcome shaped by bacterial physiology, environmental stress, and the methods used for detection.

This dissociation between culturability and viability is consistent with the adaptive biology of MTB. Under conditions such as nutrient limitation, hypoxia, antimicrobial exposure, oxidative or nitrosative stress, and host immune pressure, bacilli may reduce replication, remodel metabolism, and enter non-growing or slowly replicating states that are poorly captured by conventional culture systems (Wayne and Sohaskey, 2001; Salina et al., 2019). Importantly, the inability to initiate growth under standard laboratory conditions does not necessarily indicate a permanent loss of survival capacity. In some contexts, growth competence can be restored when environmental conditions change or when culture systems better support resuscitation (Mukamolova et al., 2010; Saito et al., 2021). Thus, the central question is not simply whether MTB is culturable or non-culturable, but how different physiological states influence the probability of bacterial recovery under specific experimental or clinical conditions.

Against this background, terminology derived from the viable but non-culturable (VBNC) concept in environmental and food microbiology (Liu et al., 2023), has been cautiously applied in tuberculosis research to describe the instance where MTB escapes recovery by standard culture while retaining molecular or metabolic characteristics that are inconsistent with irreversible cellular death, without implying a discrete or stable physiological state (Dusthackeer et al., 2019; Yeware et al., 2019; Mishra and Saito, 2022). Recently, the term differentially culturable tubercle bacilli (DCTB) has been increasingly adopted to emphasize that apparent non-culturability often reflects context-dependent limitations of detection rather than absolute loss of growth potential (Chengalroyen et al., 2016; Dusthackeer et al., 2019). Despite difference in origin and emphasis, the VBNC-like and differentially culturable phenotypes converge on a common implication: culturability is a context-dependent expression of bacterial physiology rather than a definitive indicator of survival.

The discourse surrounding VBNC state in tuberculosis has often been framed in binary terms, focusing on whether VBNC represents a distinct biological state. Such framing may obscure a more fundamental issue: MTB viability is better understood as a continuum of physiological states characterized by non-genetic heterogeneity in metabolic activity, replication competence, stress tolerance, and responsiveness to environmental stimuli (Manina et al., 2015). Within this continuum, VBNC-like and differentially culturable populations can be viewed as detection-defined manifestations of underlying bacterial heterogeneity rather than exceptional or anomalous survival forms. This conceptual shift is not merely semantic, as the way viability is defined directly affects the interpretation of diagnostic results, treatment response, and post-treatment outcomes. Culture-negative respiratory samples may still contain transcriptionally active or metabolically responsive bacilli (Zainabadi et al., 2022), treatment-induced culture conversion does not necessarily equate to a sterilizing cure (Beltran et al., 2020), and disease relapse can occur despite extended periods of culture negativity (Heyckendorf et al., 2022). These observations do not by themselves prove sustained transmissibility or inevitable treatment failure, while they highlight the limitations of relying solely on standard culture to define bacterial viability in clinical settings.

In summary, these considerations necessitate a critical reevaluation of how MTB viability is defined, measured, and interpreted. Rather than asking only whether MTB enters a singular VBNC state, a more productive approach is to examine how VBNC-like and differentially culturable phenotypes collectively reveal the conceptual and methodological limitations of culture-based definitions of survival. In this review, we integrate current evidence supporting a spectrum-based perspective of MTB viability, analyze the biological and methodological foundations underlying VBNC-like and differentially culturable phenotypes, and discuss their implications for diagnostics, treatment monitoring, and future methodological development. By integrating these phenomena within a unified viability-continuum framework, we aim to move beyond culture positivity as the sole operational criterion for viable MTB and to provide a conceptual basis that more accurately reflects the complex biology of MTB in clinical disease.

## 2 Conceptual evolution of culturability in MTB

### 2.1 VBNC concepts in tuberculosis

The concept of the viable but non-culturable (VBNC) state was originally developed in environmental and food microbiology to describe bacterial populations that retain biological viability but fail to form colonies under standard laboratory conditions (Xu et al., 1982; Oliver, 2010). Traditionally, VBNC was invoked to explain the discrepancies between culture-based enumeration and culture-independent indicators of survival, including metabolic activity or membrane integrity. Its application to MTB, however, requires reinterpretation as MTB differs fundamentally from the fast-growing bacteria in which the concept was first defined (Lipworth et al., 2016). Unlike many environmental bacteria, MTB is intrinsically slow-growing and displays stress-adaptive growth restriction as a core feature of its biology (Wayne and Sohaskey, 2001; Gengenbacher and Kaufmann, 2012; Baker et al., 2019). Therefore, the application of the VBNC concept to MTB requires more than a direct transfer of terminology, as it necessitates careful discrimination between true loss of recoverability, delayed growth, altered culture requirements, and reversible growth restriction. Early uses of VBNC-related terminology in mycobacterial research were thus primarily descriptive, referring to bacilli with evidence of biological activity that were not recovered by conventional culture, rather than establishing a novel, sharply bounded physiological state (Shleeva et al., 2004).

Importantly, the use of VBNC terminology in tuberculosis research has generally been cautious and often qualified (Shleeva et al., 2002; Li et al., 2014), with many studies favoring terms such as non-culturable dormant bacilli, differentially culturable tubercle bacilli, or differentially detectable MTB to avoid implying a uniform and stable VBNC state. Non-culturability in MTB is increasingly recognized as an operationally defined phenotype, reflecting the failure of bacilli to grow under a given set of culture conditions rather than an absolute absence of viability (Salina et al., 2014). In this review, we use “VBNC-like” as an operational descriptor for MTB populations that remain biologically active but are not recovered by standard culture, rather than as evidence for a discrete physiological category. Furthermore, dormancy is used here as a broader physiological term for growth-restricted MTB, including non-replicating, slowly replicating, or metabolically constrained bacilli, depending on the experimental or host-associated context. Dormancy should not be treated as synonymous with VBNC-like or DCTB phenotypes, because dormant bacilli may remain recoverable under some culture conditions. By contrast, VBNC-like specifically denotes the mismatch between preserved biological activity and failure of recovery by standard culture, whereas DCTB refers to bacilli that become detectable only when culture conditions are modified or made more permissive. These distinctions provide the terminology used throughout this review and are summarized in **Table 1**.

**Table 1:** Conceptual comparison of dormant MTB, VBNC-like MTB, and DCTB within a viability-continuum framework.

Feature	Dormant MTB	VBNC-like MTB	DCTB
Conceptual nature	Physiological adaptation	Descriptive and interpretative phenotype	Operational and detection-defined category
Main emphasis	Reduced replication and metabolic adaptation under stress	Retained viability despite failure of standard culture recovery	Conditional recovery under modified or permissive culture conditions
Relationship to culturability	May remain culturable despite slow or non-replicating growth	Poorly culturable or non-culturable under standard conditions	Underestimated by standard culture but recoverable by alternative assays
Typical evidence	Hypoxia-, starvation-, acid-, immune-, or drug-induced growth restriction	Molecular, metabolic, or functional indicators inconsistent with irreversible death	Liquid MPN, culture filtrate supplementation, Rpf-based recovery, or other permissive assays
Biological implication	Represents a broad stress-adapted physiological state	Indicates dissociation between viability and standard culturability	Demonstrates assay-dependent growth recovery and detection sensitivity
Interpretative limitation	Does not necessarily imply culture negativity	Should not be regarded as a discrete or stable state	Does not define a uniform physiological identity
Position in viability continuum	Broad region of reduced growth and metabolic adaptation	Viable but growth-restricted populations below standard culture thresholds	Experimentally recoverable subset of growth-restricted populations
Clinical interpretation	May contribute to persistence and treatment tolerance	May explain culture-negative but biologically active MTB signals	Complementary microbiological indicator, not a validated surrogate for relapse, transmission, or treatment failure

### 2.2 Differential culturability as an operational framework

As methodologies for recovering growth-restricted MTB evolved, particularly through liquid most-probable-number assays, culture filtrate supplementation, and resuscitation-promoting factor-based approaches (Almeida et al., 2020; Gordhan et al., 2022), the DCTB have emerged as an alternative framework for describing bacilli whose recovery depends on specific culture conditions (Beltran et al., 2022; Peters et al., 2023). DCTB are typically defined not by a unique molecular signature or a single physiological mechanism, but by their differential recovery across culture systems. In this sense, DCTB operationalizes culturability as an assay-dependent outcome influenced by both bacillary physiology and the growth-permissiveness of the culture environment.

Operationally, DCTB are commonly quantified using liquid limiting dilution or MPN-based approaches, in which serially diluted clinical specimens are incubated in liquid media with or without growth-stimulatory supplements such as MTB culture filtrate or resuscitation-promoting factors (Chengalroyen et al., 2016; Gordhan et al., 2022; Peters et al., 2023). These assays can detect bacilli that are missed or underestimated by standard CFU-based culture, thereby revealing growth-restricted populations in sputum and other patient-derived specimens (Chengalroyen et al., 2016; McAulay et al., 2018; Dusthackeer et al., 2019). Subsequent clinical studies have applied DCTB measurements during anti-tuberculosis therapy, compared DCTB readouts with routine solid and liquid culture, and evaluated their potential as complementary indicators of treatment response (Almeida et al., 2020; Beltran et al., 2022; Peters et al., 2023).

A major advantage of the DCTB framework is that it avoids assigning a fixed biological identity to all bacilli that are poorly recovered by standard culture. Instead, it offers a practical way to describe bacillary populations that become detectable under modified culture conditions. This distinction is important as differential culturability may arise from multiple, potentially overlapping mechanisms, including altered metabolic activity, delayed growth initiation, dependence on extracellular growth factors, antimicrobial exposure, or stress-induced physiological heterogeneity. Compared with VBNC terminology, DCTB is therefore more explicitly assay-defined and less dependent on assuming a discrete bacterial state.

### 2.3 VBNC-like and DCTB: detection-defined perspectives

Although VBNC-like and DCTB terminology originate from distinct conceptual traditions, they describe overlapping but not interchangeable perspectives on growth-restricted MTB populations. Both terms address bacilli that retain evidence of viability despite poor or absent recovery by standard culture, but they differ in emphasis. VBNC-like terminology is primarily interpretive, highlighting the persistence of biological activity when detectable growth is absent (Nikitushkin et al., 2021). In contrast, the DCTB framework is explicitly operational and method-oriented, referring to bacilli that become recoverable only under modified or more permissive culture conditions.

The relationship between VBNC-like and DCTB phenotypes is therefore best understood as overlapping rather than hierarchical or sequential. Many bacilli described as DCTB would satisfy broad operational definitions of VBNC-like MTB, because they remain undetected by standard culture but can be recovered under alternative conditions. However, the converse does not necessarily hold: VBNC-like populations may be inferred from molecular, metabolic, or viability-associated indicators without direct demonstration of subsequent growth recovery (Trutneva et al., 2020; Nikitushkin et al., 2021). Thus, DCTB can be viewed as the experimentally recoverable fraction of VBNC-like or culture-refractory populations, rather than as a distinct physiological category.

**Figure 1:**
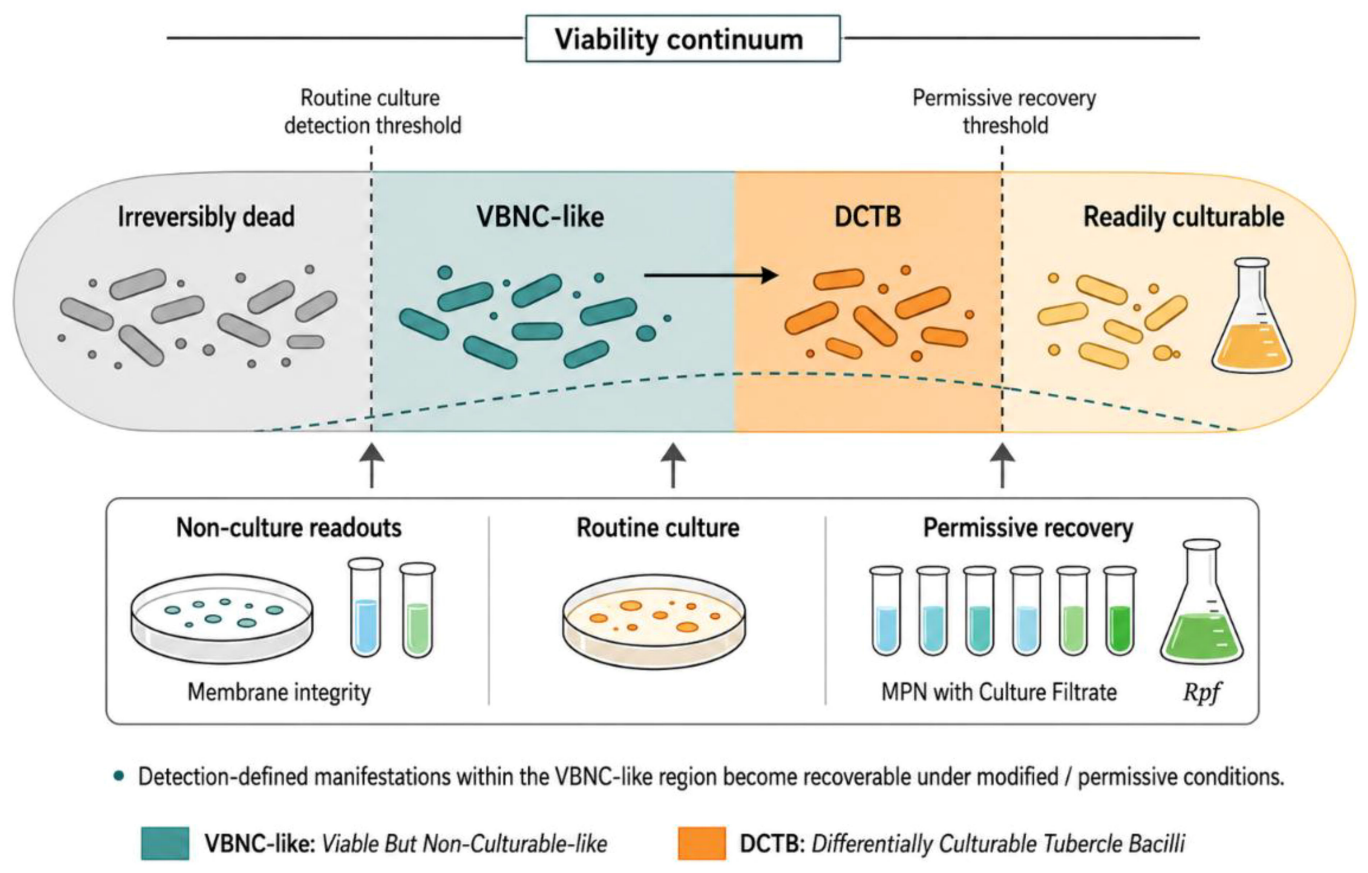
Conceptual framework of MTB viability beyond culturability: the relationship between VBNC-like and differentially culturable bacilli. Conventionally culturable MTB, VBNC-like MTB, and DCTB represent overlapping, detection-defined categories within a viability continuum. VBNC-like MTB is not recovered by standard culture, whereas DCTB becomes detectable under more permissive culture conditions.

This distinction is summarized in **Figure 1**, where VBNC-like populations are positioned as a broader conceptual category of viable but poorly culturable MTB, whereas DCTB denotes the subset that becomes recoverable under permissive culture conditions (Turapov et al., 2014). This detection-defined framework emphasizes that the distinction between VBNC-like and DCTB is primarily methodological rather than ontological. To further distinguish dormancy, VBNC-like phenotypes, and DCTB within the proposed viability-continuum framework, their conceptual and methodological differences are summarized in **Table 1**.

## 3 VBNC-like and DCTB as manifestations of a continuum of MTB viability

### 3.1 From binary viability to a continuum model in MTB

Traditional microbiological paradigms have often relied on binary classifications of bacterial survival, particularly distinctions between viable and non-viable or culturable and non-culturable populations. Although these categories provide operational clarity, they are insufficient to capture the physiological complexity of MTB during infection and treatment. This limitation is particularly evident because MTB growth, metabolism, and stress responsiveness are not fixed properties but vary across environmental, host-associated, and antimicrobial conditions (Ehrt et al., 2018). As a result, culture recovery alone cannot fully define viability, especially when bacilli retain biological activity but fail to initiate detectable growth within the constraints of standard culture assays (Sarathy, 2024).

Accumulating evidence indicates that MTB populations span a range of growth and replication competencies, from actively dividing bacilli to slowly replicating, non-replicating, or drug-tolerant subpopulations. Single-cell analyses have shown that mycobacterial replication dynamics can vary substantially within a population, supporting the view that growth competence is heterogeneous rather than uniform (Mouton et al., 2016). Similarly, metabolic heterogeneity and pathway-level noise can contribute to persister formation and altered drug susceptibility, further supporting a continuum-based interpretation of MTB survival states (Quigley and Lewis, 2021). These observations provide the empirical basis for a model in which VBNC-like and differentially culturable phenotypes are interpreted as detection-defined positions along a spectrum rather than as discrete biological states.

Conceptually, this continuum can be resolved along multiple partially independent physiological axes, including cellular integrity and survival potential, metabolic and transcriptional activity, and the capacity to initiate detectable growth within a defined assay window. These axes are not necessarily concordant: a bacillus may retain metabolic activity or stress responsiveness while failing to generate visible growth under standard culture conditions. Accordingly, discordance between culture-based and non-culture-based readouts should not be treated simply as experimental inconsistency, but as evidence that different assays interrogate different dimensions of viability (Sarathy, 2024).

Within this framework, delayed growth, dependence on growth-promoting signals, culture filtrate responsiveness, and drug-induced metabolic reprogramming can be interpreted as quantitative shifts in physiological capacity rather than qualitative transitions between live and dead states. Drug exposure can reshape MTB metabolic states and generate phenotypically tolerant subpopulations without necessarily implying irreversible loss of viability (Mishra et al., 2019; Lee et al., 2025). This continuum model therefore provides a conceptual basis for integrating VBNC-like and DCTB phenotypes into a unified understanding of MTB viability.

### 3.2 Physiological continuity decoupling viability from culturability in MTB

Loss of culturability in MTB can occur despite preservation of specific molecular and physiological functions. In dormant or non-culturable models, bacilli may retain low-abundance but stable mRNA pools, indicating that failure to form colonies does not necessarily coincide with complete transcriptional extinction (Ignatov et al., 2015). This observation provides a molecular basis for interpreting culture negativity as reduced growth recoverability rather than as direct evidence of physiological collapse. Growth-restricted MTB also undergoes organized transcriptional and metabolic remodeling under stress. In lipid-rich dormancy models, RNA-seq analyses have identified changes in lipid metabolism, stress adaptation, and growth-regulatory pathways, suggesting that growth restriction involves active physiological reprogramming rather than passive shutdown (Aguilar-Ayala et al., 2017). Similarly, nitrite-induced non-cultivability has been associated with preserved physiological responsiveness despite markedly reduced culture recovery, supporting the view that non-culturability can reflect a constrained but viable physiological condition (Gample et al., 2019).

Antimicrobial exposure provides another context where viability and culturability can become uncoupled. Drug treatment can reshape MTB metabolic states, redox balance, and stress responses, allowing subpopulations to survive transiently without acquiring genetic resistance (Mishra et al., 2019; Lee et al., 2025). In such settings, reduced culturability may reflect delayed growth initiation, altered metabolic readiness, or increased dependence on permissive recovery conditions rather than irreversible loss of survival capacity.

Resuscitation further supports physiological continuity between poorly culturable and growth-competent states. Dormant non-culturable MTB can undergo rapid transcriptional reactivation during resuscitation, indicating that return to detectable growth involves re-engagement of preserved biological potential rather than recovery from a fully inactive state (Salina et al., 2019). Together, these findings show that culturability is only one threshold-dependent readout of growth competence, whereas MTB viability may persist across multiple molecular and physiological dimensions.

### 3.3 VBNC-like and DCTB as detection-defined outcomes of the same continuum

Within the viability-continuum framework, apparent loss of culturability can be understood as a reduction in growth recoverability under a given assay condition rather than as a categorical transition to non-viability. Bacilli positioned below the threshold for growth initiation in standard culture may still retain molecular, metabolic, or stress-responsive features consistent with survival. When such populations are inferred through non-culture-based evidence of biological activity, they are commonly described as VBNC-like. When residual growth competence becomes detectable under modified or more permissive culture conditions, they are operationally classified as DCTB (Mcaulay et al., 2018; Dusthackeer et al., 2019). This framing clarifies why VBNC-like and DCTB phenotypes should be regarded as overlapping detection-defined outcomes rather than separate biological entities. The distinction between them lies primarily in the route of detection: VBNC-like terminology emphasizes preserved viability despite standard culture failure, whereas DCTB terminology requires experimental recovery under alternative culture conditions. Thus, the same underlying physiological heterogeneity may be described differently depending on whether viability is inferred from molecular or metabolic signals, or demonstrated through conditional growth recovery.

Importantly, a continuum-based interpretation does not require VBNC-like populations to progress into DCTB, or DCTB to represent a distinct downstream state. Instead, both terms mark assay-dependent positions within a heterogeneous distribution of MTB physiological capacity. This view reconciles culture-negative yet biologically active bacilli, differentially recoverable bacilli, and delayed-growth populations within a single framework, while avoiding the assumption that each detection phenotype corresponds to a discrete or stable bacterial state.

### 3.4 Critical perspective on the viability continuum

Although the viability-continuum framework offers a useful means of interpreting the dissociation between culturability and biological activity in MTB, it should be viewed as an interpretative model rather than a rigid classification system. This framework does not imply that MTB populations can currently be assigned to fixed viability stages, nor does it suggest that standardized thresholds exist for distinguishing low viability, conditional growth competence, and irreversible loss of survival potential. Rather, its principal value lies in explaining why culture-based, resuscitation-based, and molecular readouts may diverge, as these approaches interrogate different dimensions of bacterial physiology.

Alternative explanations should also be considered when interpreting culture-negative but molecularly or metabolically positive findings. Residual DNA, stable RNA species, proteins, lipids, or cell-wall components may persist after recoverable growth has been lost, and some detected signals may originate from damaged or dying bacilli rather than from organisms capable of renewed replication. Similarly, detection of DCTB may reflect the increased permissiveness of specific assay conditions rather than the existence of a discrete *in vivo* bacterial state. Therefore, continuum-based interpretations should not be used to overstate the clinical significance of every non-culture-positive signal. Future studies incorporating standardized assays and longitudinal clinical outcomes are needed to determine which dimensions of this continuum have diagnostic or prognostic relevance.

## 4 Resuscitation and its interpretative limits

### 4.1 Resuscitation: observations and pitfalls

**Figure 2:**
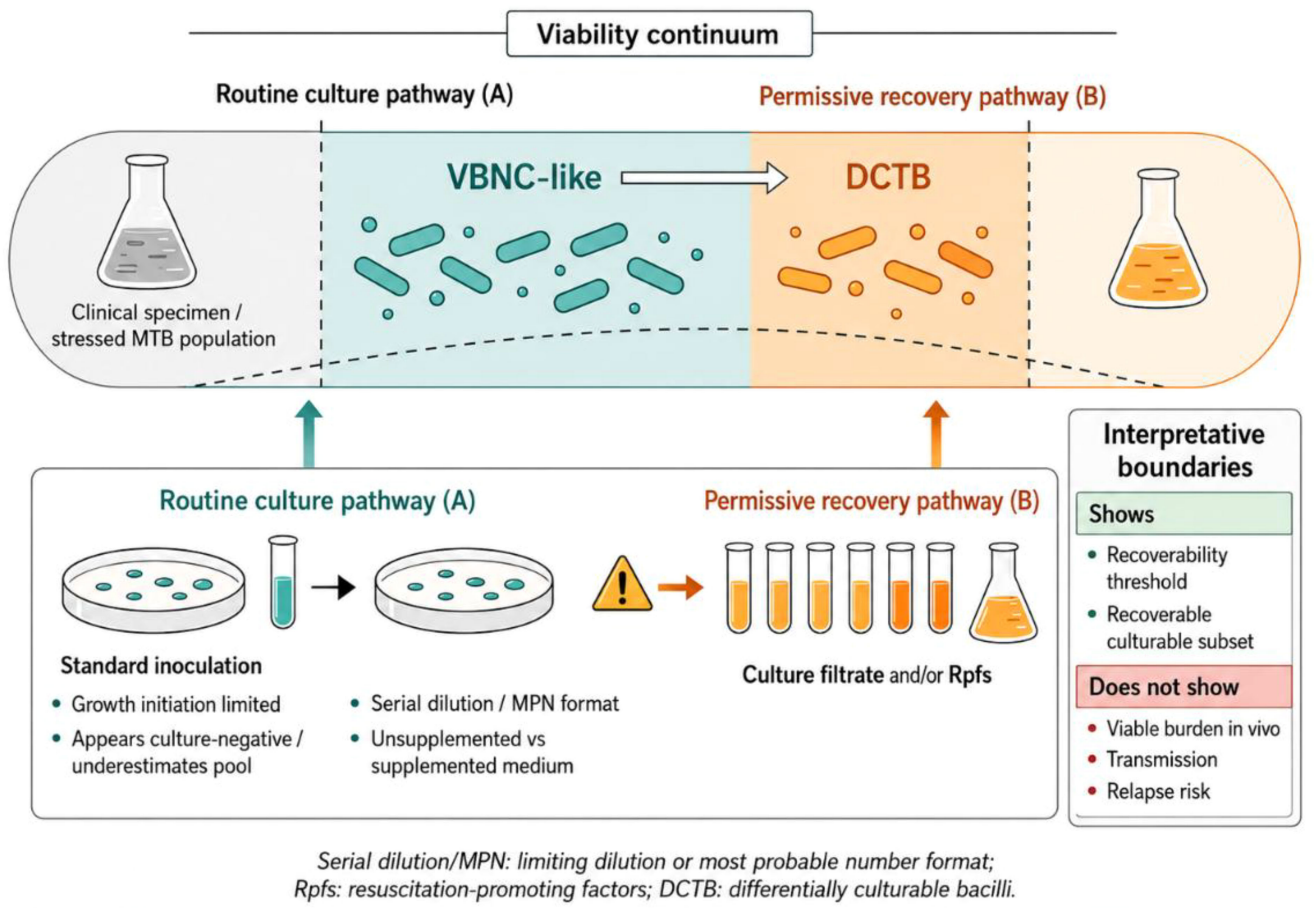
How resuscitation-based assays reveal differential culturability. Standard CFU culture detects colony-forming bacilli, whereas liquid MPN, culture filtrate, or Rpf-based assays may recover additional growth-restricted MTB. Such recovery reflects assay-accessible growth potential rather than a distinct dormant state.

Resuscitation has played a central role in shaping discussions of non-culturable and growth-restricted MTB populations. A key observation is that bacilli missed by standard CFU-based culture can, in some settings, be recovered under more permissive conditions. Resuscitation-based assays therefore provide a useful experimental illustration of conditional recoverability. As shown in **Figure 2**, standard CFU-based culture detects bacilli that initiate growth under conventional conditions, whereas limiting-dilution or MPN-based assays supplemented with culture filtrate or resuscitation-promoting factors may recover additional growth-restricted bacilli.

However, this increased recovery should be interpreted as an expansion of assay sensitivity and growth permissiveness, rather than as definitive evidence that the recovered bacilli previously occupied a single, uniform dormant state (Veatch and Kaushal, 2017; Saito et al., 2021). Growth under Rpf- or culture-filtrate-supplemented conditions demonstrates that at least some bacilli retain residual growth competence, but it does not establish that all recovered bacilli shared the same physiological identity before resuscitation. Culture filtrate is a complex supplement, and recovery outcomes can also be influenced by inoculum size, bacillary aggregation, sample processing, incubation time, and endpoint criteria (Chengalroyen et al., 2022).

Accordingly, the interpretative value of resuscitation lies not in defining a discrete survival state, but in revealing the conditional nature of MTB recoverability. Bacilli that fail to grow on standard solid media may remain below the detection threshold of conventional culture yet become detectable when growth-promoting signals, liquid culture formats, or extended incubation conditions are provided. Resuscitation therefore provides a powerful tool for uncovering growth-restricted bacilli, while also highlighting the methodological limits of using growth recovery alone to define viability.

### 4.2 Molecular basis of Rpf-mediated growth recovery

Although resuscitation-promoting factors (Rpfs) are important mediators of growth recovery in some non-culturable or growth-restricted mycobacterial populations, the precise molecular mechanisms through which they stimulate resuscitation remain incompletely defined. MTB encodes five Rpf-like proteins, RpfA-RpfE, which contain conserved catalytic domains related to peptidoglycan-hydrolyzing enzymes. Structural and biochemical studies indicate that these domains share features with lysozyme-like or lytic transglycosylase-like enzymes, supporting a model in which Rpfs promote growth re-entry by locally remodeling peptidoglycan and facilitating cell wall expansion (Cohen-Gonsaud et al., 2005; Bavesh and Mizrahi, 2010; Ruggiero et al., 2016). This cell wall-centered model provides a plausible biochemical explanation for Rpf-dependent growth recovery, but it should not be interpreted as a complete mechanism of resuscitation. Rpf-mediated peptidoglycan cleavage may generate structural changes or muropeptide signals that lower the threshold for renewed growth, yet recovery is likely to depend on additional factors, including metabolic readiness, cell envelope integrity, stress history, and the permissiveness of the surrounding culture environment (Nikitushkin et al., 2015). Thus, Rpfs are best regarded as contributors to growth re-entry rather than as sole determinants of viability.

Expression studies further suggest that individual *rpf* genes are regulated in a condition-dependent manner. In comparative expression analyses, *rpfA* and *rpfD* were associated with early resuscitation, *rpfC* expression was maintained under nutrient stress, whereas acid stress and hypoxia were associated with increased expression of *rpfD*/*rpfE* and *rpfC*/*rpfE*, respectively (Gupta et al., 2010; Wivagg and Hung, 2012). These patterns support the possibility of partial functional specialization among Rpf homologs, although the extent to which each Rpf contributes to specific host-associated or experimental growth restrictions remains context dependent.

Genetic studies also indicate that Rpfs are not strictly required for replication under nutrient-rich laboratory conditions. Strains lacking multiple *rpf* genes can retain growth capacity in permissive media but show defects in resuscitation, stress adaptation, cell envelope-associated functions, or virulence-associated phenotypes. These observations support the interpretation that Rpfs modulate the probability of growth re-entry under restrictive conditions rather than defining viability itself (Kana et al., 2008; Wivagg and Hung, 2012). Collectively, Rpf-mediated resuscitation is best viewed as a conditional restoration of growth competence when cell wall, metabolic, and environmental constraints become permissive for renewed replication.

### 4.3 Resuscitation as a detection-dependent outcome

Quantitative comparisons between CFU-based culture and limiting-dilution or MPN-based recovery assays provide a direct way to evaluate the threshold-dependent nature of MTB recoverability. In clinical sputum samples, DCTB detected by limiting-dilution assays supplemented with culture filtrate have been reported to exceed colony-forming units (CFU) by 10¹~10³-fold, exhibiting considerable variability among patients (Mcaulay et al., 2018; Mesman et al., 2021). This broad distribution indicates that standard CFU-based culture does not capture a fixed fraction of viable bacilli, but instead imposes an assay-specific threshold for growth detection.

Importantly, differential detectability is not confined to post-treatment samples. Studies of pretreatment or treatment-naïve sputum have shown that differentially detectable MTB can be present before antimicrobial exposure, indicating that altered culturability may arise naturally during infection rather than being solely treatment-induced. Treatment may further reshape this distribution by reducing readily culturable bacilli while enriching or revealing populations with delayed or conditional growth recovery.

Within a continuum-based framework, successful resuscitation should therefore be interpreted as assay-accessible growth potential rather than as a categorical transition between distinct survival states. Conversely, failure to recover bacilli under a given resuscitation protocol does not necessarily indicate non-viability as it may not provide the specific signals, incubation window, or environmental permissiveness required by particular growth-restricted subpopulations. Resuscitation is therefore best understood as a detection-dependent outcome shaped collectively by bacterial physiological state and assay design.

### 4.4 Methodological boundaries of resuscitation assays

Taken together, resuscitation-based approaches are best understood as assay modifiers that increase growth permissiveness and detection sensitivity beyond the limits of standard culture. Their main value lies in testing whether culture-negative or poorly culturable samples contain bacilli with residual growth potential under defined permissive conditions. Operationally, resuscitation assays extend the framework introduced for differential culturability, while also imposing their own interpretative boundaries. The information provided by these assays remains methodologically constrained. Quantitative sputum studies have shown that Rpf or culture-filtrate supplementation can reveal bacillary populations missed or underestimated by CFU-based culture. These findings indicate that resuscitation assays measure conditional recoverability rather than absolute viability or total bacterial burden. A positive result demonstrates assay-accessible growth potential, but it does not establish the precise physiological state of bacilli *in vivo*. Conversely, a negative result does not necessarily prove non-viability, because the assay may fail to provide the specific signals, incubation window, or environmental permissiveness required by particular growth-restricted subpopulations.

Accordingly, Rpf-based resuscitation assays are useful for investigating assay sensitivity, constraints on growth initiation, and differential recoverability. They are insufficient on their own to infer *in vivo* physiological transitions, accurately estimate total bacterial burden within the host, or predict disease progression, transmission, relapse, or treatment outcome. This methodological boundary preserves the explanatory value of resuscitation assays while preventing the overextension of growth-recovery data into unsupported biological or clinical claims.

By framing resuscitation as an assay-conditioned readout, this section narrows the interpretation of culture negativity to a methodological question: what forms of growth potential are detectable under the conditions provided? This perspective provides a transition to the next section, where the clinical implications of culture negativity can be considered without treating standard culture, resuscitation, or DCTB recovery as definitive measures of bacterial fate.

## 5 Clinical implications beyond culturability

### 5.1 The culture-negative paradox

Culture-based methods remain indispensable in tuberculosis diagnosis and treatment monitoring as they provide direct evidence of bacilli capable of replication under defined laboratory conditions. However, clinical practice frequently reveals discordance between culture results and other disease-associated readouts, including nucleic acid-based assays, host-response markers, radiographic abnormalities, and clinical trajectories (Mtafya et al., 2022). Such discordance does not necessarily indicate methodological inconsistency. Instead, it reflects the fact that different assays interrogate different biological dimensions of tuberculosis: culture detects recoverable growth, molecular assays detect bacterial nucleic acids or transcriptional signals, host-response markers capture immune activation, and imaging reflects tissue-level pathology (Heyckendorf et al., 2022).

The key interpretative challenge is that culture negativity is often treated as a proxy for bacterial clearance, whereas non-culture positivity is sometimes interpreted as evidence of persistent viable infection (Shubhada et al., 2016). A negative culture result indicates the absence of detectable growth under the applied conditions, but it does not exclude the presence of bacilli or bacterial remnants that remain below the threshold for culture recovery. Conversely, persistent molecular, immunological, or radiographic signals do not by themselves prove ongoing replication, transmissibility, treatment failure, or future relapse (Malherbe et al., 2016). They may reflect residual bacterial material, low-level or spatially restricted bacterial activity, delayed clearance of host inflammatory responses, or tissue damage that persists after loss of recoverable growth.

Within this clinical context, VBNC-like and differentially culturable phenotypes provide one possible biological explanation for culture-negative but signal-positive findings. Growth-restricted bacilli may retain molecular or metabolic features that are detectable by non-culture approaches while remaining inaccessible to standard culture systems. However, these phenotypes should be interpreted as part of a broader diagnostic mismatch rather than as proof that every culture-negative, non-culture-positive sample contains viable MTB (Alebouyeh et al., 2022). The clinical significance of such findings depends on the type of signal detected, the timing of sampling, treatment status, disease site, and the patient’s immunological and radiographic context.

**Figure 3:**
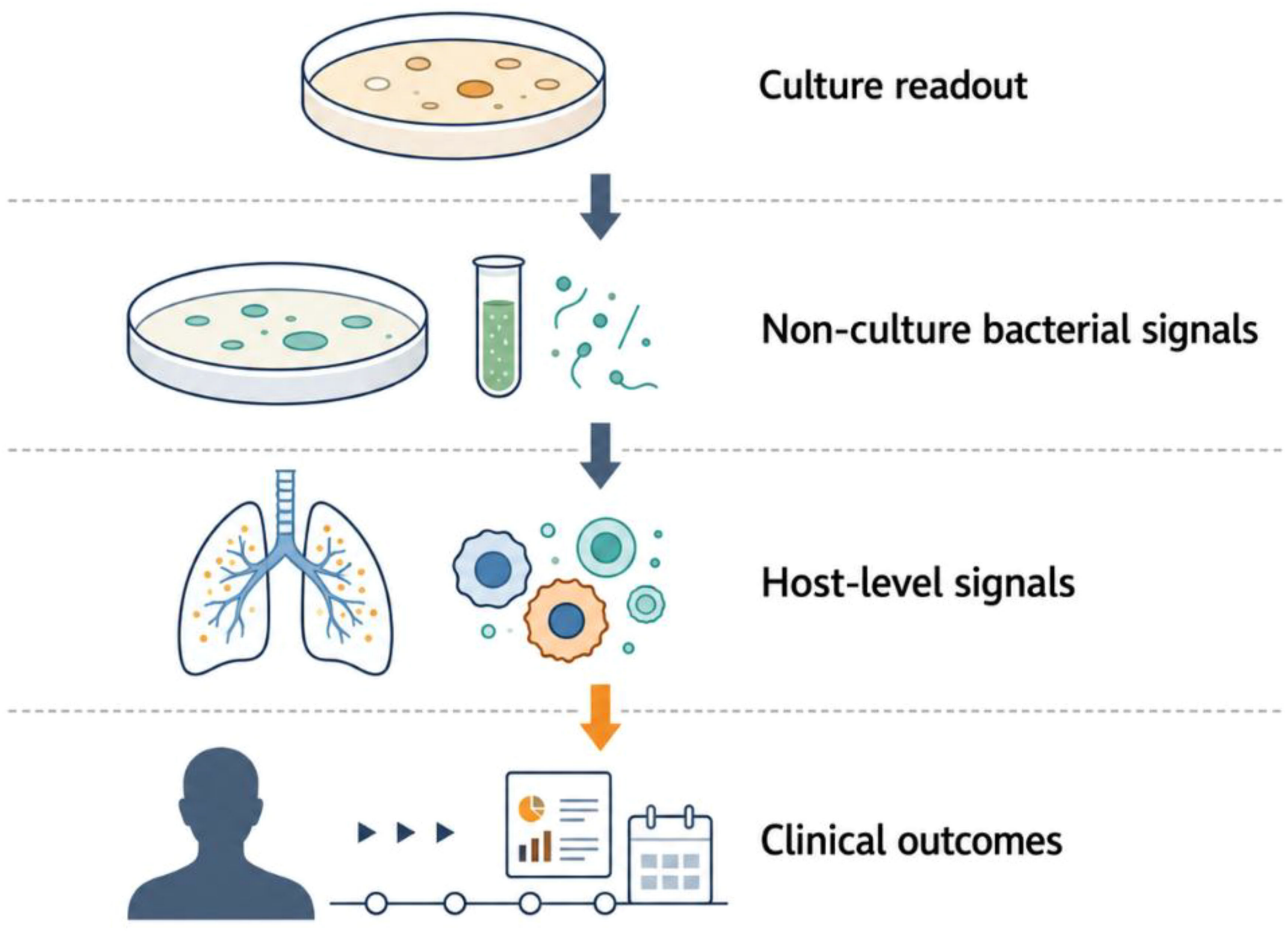
Layers of clinical interpretation beyond culture detection. Culture, molecular assays, host-response markers, imaging, and clinical findings reflect different dimensions of TB biology. These readouts should be interpreted as complementary rather than interchangeable indicators of viable MTB.

These discrepancies indicate that culture detection, bacterial viability, growth competence, and clinical relevance should not be treated as interchangeable concepts. A layered interpretative model is therefore needed to distinguish what each diagnostic or clinical readout can and cannot infer. As summarized in **Figure 3**, culture negativity primarily reflects the absence of recoverable growth under defined laboratory conditions, whereas molecular, metabolic, immunological, radiographic, and clinical indicators may capture different dimensions of MTB persistence or disease activity. This framework allows culture-negative results to be interpreted more cautiously, without dismissing non-culture signals as irrelevant or overinterpreting them as definitive evidence of ongoing viable infection.

### 5.2 Viability, growth competence and treatment response

A key clinical implication of VBNC-like and differentially culturable MTB populations is the need to distinguish bacterial viability, growth competence, and clinical significance. Physiological viability does not necessarily correspond to the ability to replicate under standard culture conditions, and loss of recoverable growth does not by itself establish sterilizing cure. This distinction is consistent with tuberculosis pharmacology, where bactericidal activity and sterilizing activity represent related but non-identical dimensions of treatment efficacy (Mitchison and Davies, 2012; Davies, 2014). Host immunity, anatomical localization, antimicrobial exposure, lesion microenvironments, and sampling time all influence whether bacilli with residual viability have measurable clinical significance.

Culture conversion should therefore be interpreted as a clinically important but incomplete marker of treatment response. In practical terms, conversion from culture-positive to culture-negative sputum indicates that bacilli capable of growth under the applied laboratory conditions are no longer detected in the sampled compartment. However, it does not prove that all physiologically intact or growth-restricted bacilli have been eliminated from the host. This distinction helps explain why sputum culture conversion remains useful for treatment monitoring while showing variable predictive value for relapse, treatment failure, or final outcome across patient populations, disease forms, and assessment timepoints (Kurbatova et al., 2015; Phillips et al., 2016; Calderwood et al., 2021).

Accordingly, culture conversion should be viewed as one layer of treatment-response assessment rather than as a definitive measure of bacterial fate. Bacterial material or physiologically constrained bacilli may persist below the threshold of routine culture detection without necessarily implying imminent disease progression. Conversely, delayed or absent culture conversion remains clinically important because it may reflect high bacillary burden, inadequate drug exposure, drug resistance, extensive disease, or impaired host control. This layered interpretation preserves the clinical value of culture conversion while acknowledging that treatment response is shaped by heterogeneous MTB survival states, host-pathogen interactions, and the methodological limits of culture-based monitoring.

### 5.3 Therapeutic implications of growth-restricted MTB

Recognition of growth-restricted and differentially culturable MTB populations has important implications for interpreting therapeutic efficacy. These populations highlight a central pharmacological issue: bacilli that are poorly recovered by standard culture may remain biologically vulnerable to interventions that disrupt essential survival functions or modify the host environments that support persistence. Accordingly, treatment effects should not be interpreted solely through the lens of active replication or conventional culture recovery. Several antimicrobial strategies illustrate this principle. Bedaquiline targets mycobacterial ATP synthase and disrupts ATP homeostasis, providing a rationale for activity against metabolically constrained or dormant mycobacteria in addition to actively replicating bacilli (Koul et al., 2008; Kiel et al., 2015). Similarly, nitroimidazole-class agents such as pretomanid, formerly PA-824, require intracellular reductive activation and can affect nonreplicating MTB through mechanisms that include nitric oxide release and respiratory poisoning under hypoxic or nonreplicating conditions (Manjunatha et al., 2006; Singh et al., 2008). These examples indicate that non-replication does not necessarily imply pharmacological inaccessibility.

Drug exposure may also alter the distribution of bacillary phenotypes detected by different culture methods. Clinical and experimental studies have shown that differentially detectable or differentially culturable MTB populations can change during treatment, and recent experimental work suggests that drug exposure itself can induce differential culturability in diverse MTB strains (March et al., 2025; Mcaulay et al., 2018). Such findings support the interpretation that therapeutic pressure may reshape recoverability profiles, even when standard culture readouts provide only a partial view of bacterial response. On the other hand, host-directed approaches add another layer to this interpretation. Rather than directly killing bacilli, these interventions aim to alter host pathways that influence intracellular survival, immune control, or lesion microenvironments. Metformin, for example, has been reported to restrict intracellular MTB growth through host metabolic and immune pathways involving AMPK-related signaling, mitochondrial reactive oxygen species, and macrophage effector functions in experimental and translational settings (Singhal et al., 2014; Kim et al., 2020). These observations suggest that host-directed interventions may affect the conditions under which growth-restricted bacilli persist, although their effects on VBNC-like or DCTB populations in patients remain incompletely defined.

These therapeutic examples should be interpreted within well-defined experimental and clinical limits. They do not provide direct evidence that VBNC-like or differentially culturable populations require changes to established treatment regimens, nor do they demonstrate altered clinical efficacy in individual patients. Instead, they emphasize that culture-based endpoints capture only part of the bacterial response to therapy. Incorporating non-growth-based or conditionally growth-based readouts may therefore help characterize treatment effects on physiologically constrained subpopulations, while avoiding the overinterpretation of culture negativity as definitive sterilizing cure.

### 5.4 Clinical interpretative limits of DCTB

DCTB assays demonstrate that standard culture can underestimate conditionally recoverable MTB populations, but DCTB detection should not be equated with active disease, transmissibility, treatment failure, or inevitable relapse. Longitudinal studies suggest that DCTB dynamics may provide additional information about treatment response, and residual DCTB has been associated with subsequent microbiological or clinical signals in some cohorts (Almeida et al., 2020; Peters et al., 2023). However, clinically actionable thresholds linking DCTB burden to relapse risk, transmission, or regimen selection have not yet been validated.

Therefore, DCTB should be interpreted as a complementary microbiological readout rather than a stand-alone surrogate endpoint. Its value lies in revealing growth-restricted bacilli missed by standard culture and in refining the biological interpretation of treatment response, not in independently guiding clinical decisions. Further prospective studies with standardized assays and outcome-linked thresholds are required before DCTB measurements can be incorporated into clinical treatment-monitoring frameworks.

### 5.5 Molecular viability assays in future monitoring strategies

Emerging molecular viability assays may help bridge the gap between culture-based detection and treatment-response assessment. Conventional DNA-based tests are highly valuable for diagnosis, but DNA can persist after bacillary death and therefore has limited value for distinguishing viable from non-viable MTB during therapy. RNA-based approaches, including assays targeting 16S rRNA or selected mRNA transcripts, may provide a more dynamic indication of bacterial activity. Molecular bacterial load assays and optimized sputum RNA detection methods have therefore been explored as culture-independent tools for quantifying bacillary burden and monitoring early treatment response (Honeyborne et al., 2011; Said et al., 2021). However, RNA positivity should still be interpreted cautiously, as RNA abundance may be influenced by metabolic state, antimicrobial exposure, sample processing, RNA stability, and assay sensitivity.

Other viability-associated molecular approaches, such as PMA- or EMA-qPCR, may reduce amplification from membrane-compromised bacilli and thereby improve discrimination between intact and damaged cells (Kim et al., 2014). However, their application to MTB remains technically challenging because of the lipid-rich mycobacterial cell envelope, heterogeneous dye penetration, complex sputum matrices, and incomplete suppression of residual DNA signals. Thus, these assays should be viewed as complementary indicators of bacterial activity or cellular integrity rather than definitive substitutes for culture. In future monitoring strategies, their greatest value may lie in combination with conventional culture, MGIT-based detection, resuscitation-based assays, radiographic assessment, host-response markers, and clinical evaluation. Prospective studies using standardized protocols, quantitative thresholds, and longitudinal outcome data will be required to define whether molecular viability readouts can improve relapse-risk assessment, treatment monitoring, or regimen evaluation. The principal viability assessment methods discussed in this review are summarized in **Table 2**, highlighting their main readouts, strengths, limitations, and potential applications.

**Table 2:** Summary of major methods for assessing MTB viability.

Method	Main readout	Strengths	Limitations	Practical use
Solid culture: LJ, Ogawa, 7H10/7H11	Colony formation	Established; isolate recovery; DST-compatible	Slow; misses growth-restricted bacilli	Diagnosis; CFU counting; isolate recovery
Liquid culture/MGIT	Growth in liquid medium	Faster than solid culture; clinically standardized	Contamination risk; species confirmation needed	Diagnosis; treatment monitoring; DST
CFU counting	Colony-forming bacilli	Quantitative; simple	Underestimates VBNC-like/DCTB populations	Experimental bacterial burden
MPN/limiting dilution	Conditional liquid recovery	Sensitive for low-burden or stressed bacilli	Labor-intensive; assay-dependent	DCTB quantification; persistence studies
Culture filtrate/Rpf assays	Resuscitation-dependent recovery	Reveals poorly culturable bacilli	Not a direct *in vivo* state marker	Resuscitation and differential culturability studies
RNA-based assays/MBLA	RNA-associated bacterial activity	Culture-independent; dynamic	RNA stability; not proof of replication	Early treatment-response monitoring
PMA/EMA-qPCR	Membrane integrity-associated DNA signal	Reduces dead-cell DNA signal	Dye penetration; MTB cell-wall effects	Viability-associated molecular assessment

## 6 Conclusion and perspectives

Tuberculosis research has long relied on culturability as the dominant operational readout of bacterial viability. Although growth-based assays remain crucial for diagnosis and treatment monitoring, evidence synthesized in this review demonstrates that culturability represents only a limited dimension of MTB survival. The existence of VBNC-like and differentially culturable phenotypes illustrates a persistent connect between bacterial viability and growth-based detectability, highlighting the inadequacies of equating culture negativity with the absence of bacteria.

By integrating sights from experimental models, molecular viability assays, and clinical studies, this review proposes a continuum-based interpretation of MTB viability, suggesting that bacterial populations occupy diverse physiological states rather than distinct survival states. Within this framework, apparent non-culturability emerges as a detection-defined outcome influenced by physiological constraints and methodological thresholds, rather than as evidence of irreversible bacterial death. This perspective reconciles long-standing discrepancies between culture results and biological or clinical indicators, including the culture-negative paradox observed during treatment and follow-up.

A key implication of this synthesis is the necessity to differentiate between various dimensions of bacterial survival. Physiological viability, replicative viability, and clinical relevance represent related but non-equivalent dimensions, each examined through different diagnostic or experimental methodologies. Culture-based assays predominantly assess replicative competence under standardized conditions, whereas non-culture-based methods may encompass broader aspects of biological persistence without implying disease activity or transmissibility. Moving forward, greater conceptual clarity in defining the specific dimension of viability under evaluation will be essential for aligning experimental design, diagnostic interpretation, and clinical endpoints. By reframing viability beyond culturability and emphasizing the conditional nature of growth-based detection, this review provides a coherent framework for interpreting bacterial persistence, clearance, and treatment response in tuberculosis.
